# Consensus report from the 9^th^ International Forum for Liver Magnetic Resonance Imaging: applications of gadoxetic acid-enhanced imaging

**DOI:** 10.1007/s00330-020-07637-4

**Published:** 2021-02-01

**Authors:** Dow-Mu Koh, Ahmed Ba-Ssalamah, Giuseppe Brancatelli, Ghaneh Fananapazir, M. Isabel Fiel, Satoshi Goshima, Sheng-Hong Ju, Nikolaos Kartalis, Masatoshi Kudo, Jeong Min Lee, Takamichi Murakami, Max Seidensticker, Claude B. Sirlin, Cher Heng Tan, Jin Wang, Jeong Hee Yoon, Mengsu Zeng, Jian Zhou, Bachir Taouli

**Affiliations:** 1 Department of Diagnostic Radiology, Royal Marsden Hospital, Sutton, UK; 2 Department of Biomedical Imaging and Image-Guided Therapy, Medical University of Vienna, Vienna, Austria; 3 Dipartimento di Biomedicina, Neuroscienze e Diagnostica avanzata (BiND), University of Palermo, Palermo, Italy; 4 Department of Radiology, Mayo Clinic Arizona, Phoenix, AZ USA; 5 Department of Pathology, Molecular and Cell Medicine, Icahn School of Medicine at Mount Sinai, New York, NY USA; 6 Department of Diagnostic Radiology & Nuclear Medicine, Hamamatsu University School of Medicine, Hamamatsu, Japan; 7 Department of Radiology, Zhongda Hospital, Southeast University, Nanjing, People’s Republic of China; 8 Department of Radiology Huddinge, Karolinska University Hospital, Stockholm, Sweden; 9 Division of Radiology, CLINTEC, Karolinska Institutet, Stockholm, Sweden; 10 Department of Hepatology and Gastroenterology, Kindai University Faculty of Medicine, Osaka, Japan; 11 Department of Radiology, College of Medicine, Seoul National University, Seoul, South Korea; 12 Department of Radiology, Kobe University Graduate School of Medicine, Kobe, Japan; 13 Klinik und Poliklinik für Radiologie, Klinikum der Universität München, Munich, Germany; 14 Department of Radiology, University of California San Diego, San Diego, CA USA; 15 Department of Diagnostic Radiology, Tan Tock Seng Hospital, Lee Kong Chian School of Medicine, Singapore, Singapore; 16 Department of Radiology, Third Affiliated Hospital of Sun Yat Sen University, Guangzhou, People’s Republic of China; 17 Department of Radiology, Zhongshan Hospital, Fudan University, Shanghai, People’s Republic of China; 18 Liver Cancer Institute, Zhongshan Hospital, Fudan University, Shanghai, People’s Republic of China; 19 Department of Diagnostic, Molecular, and Interventional Radiology, BioMedical Engineering and Imaging Institute, Icahn School of Medicine at Mount Sinai, New York, NY USA

**Keywords:** Gadoxetic acid, Hepatocellular carcinoma, Magnetic resonance imaging, Metastatic liver disease

## Abstract

**Objectives:**

The 9th International Forum for Liver Magnetic Resonance Imaging (MRI) was held in Singapore in September 2019, bringing together radiologists and allied specialists to discuss the latest developments in and formulate consensus statements for liver MRI, including the applications of gadoxetic acid–enhanced imaging.

**Methods:**

As at previous Liver Forums, the meeting was held over 2 days. Presentations by the faculty on days 1 and 2 and breakout group discussions on day 1 were followed by delegate voting on consensus statements presented on day 2. Presentations and discussions centered on two main meeting themes relating to the use of gadoxetic acid–enhanced MRI in primary liver cancer and metastatic liver disease.

**Results and conclusions:**

Gadoxetic acid–enhanced MRI offers the ability to monitor response to systemic therapy and to assist in pre-surgical/pre-interventional planning in liver metastases. In hepatocellular carcinoma, gadoxetic acid–enhanced MRI provides precise staging information for accurate treatment decision-making and follow-up post therapy. Gadoxetic acid–enhanced MRI also has potential, currently investigational, indications for the functional assessment of the liver and the biliary system. Additional voting sessions at the Liver Forum debated the role of multidisciplinary care in the management of patients with liver disease, evidence to support the use of abbreviated imaging protocols, and the importance of standardizing nomenclature in international guidelines in order to increase the sharing of scientific data and improve the communication between centers.

**Key Points:**

• *Gadoxetic acid*–*enhanced MRI is the preferred imaging method for pre-surgical or pre-interventional planning for liver metastases after systemic therapy.*

• *Gadoxetic acid*–*enhanced MRI provides accurate staging of HCC before and after treatment with locoregional/biologic therapies.*

• *Abbreviated protocols for gadoxetic acid–enhanced MRI offer potential time and cost savings, but more evidence is necessary. The use of gadoxetic acid–enhanced MRI for the assessment of liver and biliary function is under active investigation.*

**Supplementary Information:**

The online version contains supplementary material available at 10.1007/s00330-020-07637-4.

## Introduction

The 9th International Forum for Liver Magnetic Resonance Imaging (MRI) was held in September 2019 in Singapore and attended by 90 invited delegates from Asia (*n* = 53), Europe (*n* = 20), North America (*n* = 8), Central America (*n* = 4), South America (*n* = 2), and Australia/New Zealand (*n =* 3). The majority of delegates were radiologists with expertise in liver MRI, including the use of gadoxetic acid (Primovist, Eovist, Bayer AG); other delegates provided representation from surgery, pathology, and hepatology (see Supplement 1). Discussion and consensus voting at the Forum focused on six topics:

- Referrer focus: how to build a well-functioning multidisciplinary team in patients with liver tumors
- Abbreviated MRI protocols for evaluation of liver metastases and hepatocellular carcinoma (HCC)
- Evaluation of treatment response of liver metastases and clinical impact
- International diagnostic HCC guidelines and Liver Imaging Reporting and Data System (LI-RADS)
- Gadoxetic acid–enhanced MRI for treatment decision-making and follow-up of HCC
- Future possible indications of gadoxetic acid–enhanced MRI.

Consensus statements on these topics were generated at workshops, following peer review of the literature available at the time of the Forum. Delegate voting on each statement could be either “agree,” “disagree,” or “abstain.” A consensus on each statement was considered to be reached if at least 80% of voting delegates agreed.

In this article, we review the relevant literature published prior to and available for discussion at the Forum. Any literature published after the Forum, even if related to the topic, has not been included because it was not available contemporaneously to the delegates.

## Referrer focus: how to build a well-functioning multidisciplinary team in patients with liver tumors

The multidisciplinary team approach utilizes regular, scheduled discussions among diverse specialists to review the diagnosis, treatment, and outcomes of individual patients [1]. The adoption of multidisciplinary teams represents best practice in current standards of cancer care [2–4]. In relation to liver disease, the American Association for the Study of Liver Diseases (AASLD) states: “Hepatocellular carcinoma patients should be seen in [multidisciplinary] clinics whenever it is feasible and, if not, a referral to a center with a true multidisciplinary clinic should be considered” [5], while the LI-RADS version 2018 states: “Since radiologists may not know all relevant factors, multidisciplinary discussion for consensus-based management may be helpful in difficult cases” [6].

The exact composition of liver multidisciplinary teams and their regularity and methodology for meeting (whether physical or online) vary between institutions, and there is no single definition for what constitutes multidisciplinary care. Typically, specialists attending liver multidisciplinary teams include diagnostic and interventional radiologists, medical oncologists, radiation oncologists, hepatologists, surgeons, pathologists, and support services, reflecting the complex needs and multiple treatment options available to these patients. Radiologists’ roles include interpretation of images for diagnosis, staging, and post-treatment monitoring; performing interventional radiologic procedures; standardizing and updating institutional imaging guidelines; and exchanging experiences with other specialists. The following review of multidisciplinary teams focuses on survival as a measure for improved outcome.

Chang et al [7] and Yopp et al [8] compared two patient cohorts with newly diagnosed HCC before and after the implementation of multidisciplinary care. Median patient survival improved after multidisciplinary care implementation, at 13.2 versus 4.8 months (*p* = 0.005) in the study by Yopp et al. Another investigational approach, adopted in two retrospective studies, compared survival of cohorts who did versus those who did not receive multidisciplinary care [9, 10]. Both studies reported that multidisciplinary team review was associated with significant improvements in survival (e.g., 5-year survival 71.4% vs. 58.7%; *p* < 0.001 [10]). Finally, Charriere et al compared patient survival when the multidisciplinary team’s treatment decision was or was not followed [11]. Factors associated with a negative prognosis were (a) not following the multidisciplinary team’s treatment decision (hazard ratio [HR]: 0.39; *p* < 0.001), together with (b) elevated serum alpha-fetoprotein level (HR: 0.63; *p* = 0.005), and (c) being outside the Milan criteria for liver transplant eligibility (HR: 0.45; *p* < 0.001). Hence, despite methodologic limitations associated with retrospective studies, non-identical patient populations, and variable definitions of multidisciplinary care, these studies indicate that the multidisciplinary team approach improves survival in patients with HCC, providing the greatest benefit in patients with advanced tumor stage or more complex risk factors.

Few studies have investigated the influence of multidisciplinary care in metastatic liver disease [12]. However, the role of multidisciplinary teams is supported by multidisciplinary consensus panels that emphasize the importance of specialized, individualized patient care [13].

- *Consensus statement #1*
- To optimize patient care and improve survival, patients with suspected or diagnosed HCC should be evaluated utilizing a multidisciplinary approach (72/75; 96% agreement).
- *Consensus statement #2*
- A multidisciplinary approach is suggested for the management of patients with metastatic disease to the liver (76/79; 96% agreement).
- *Consensus statement #3*
- A radiologist should be a core member of any multidisciplinary team for HCC or metastatic liver disease (70/78; 90% agreement).

## Abbreviated MRI protocols for evaluation of liver metastases and HCC

Abbreviated MRI protocols that utilize shorter or fewer imaging sequences have been developed to reduce imaging time, patient discomfort, and potentially costs [14]. Studies of abbreviated MRI protocols cited here are classified into two categories: (a) abbreviated MRI, including hepatobiliary phase (HBP), and (b) non-contrast MRI.

### Abbreviated MRI protocols, including HBP

Three retrospective studies simulated the use of abbreviated gadoxetic acid–enhanced MRI protocols for HCC surveillance [15–17]. Marks et al reported that an abbreviated MRI protocol comprising T2-weighted (T2W) single-shot, fast spin-echo (SSFSE) and T1-weighted (T1W) HBP imaging 20-min post-gadoxetic acid administration had a negative predictive value (NPV) of 96–97% compared with the composite reference standard [15]. Inclusion of a diffusion-weighted imaging (DWI) sequence did not alter the performance of the abbreviated protocol. Tillman et al also compared T2W SSFSE and T1W HBP imaging 20-min post-gadoxetic acid injection versus a composite reference [16]. Per-lesion sensitivity and NPV of the abbreviated protocol were 85% and 95%, respectively. In the study by Besa et al, T1W HBP imaging 20-min post-gadoxetic acid injection provided per-patient sensitivity and NPV (at 90% and 94%, respectively, pooled data) equivalent to a full protocol (90% and 95%, respectively) [17]. Combining T1W HBP with DWI in the abbreviated protocol reduced sensitivity and NPV (81% and 90%, respectively).

Finally, Canellas et al assessed an abbreviated imaging protocol including ultrafast spin-echo T2W, T1W HBP 20-min post-gadoxetic acid injection, and DWI against a full protocol in patients with pathologically proven colorectal liver metastases (CRLMs) [18]. There were no statistically significant differences in sensitivity or lesion characterization (all > 90%) between the protocols.

Abbreviated protocols in three of the above studies provided cost savings of 31% [15], 31% [17], and 41% [18] versus full protocols by reducing the imaging time. Absolute Medicare reimbursement costs (2017–dated) for the abbreviated and full protocols, respectively, were $365.9 versus $527.8 [17] and $311.33 versus $528.70 [18].

### Abbreviated protocols using non-contrast MRI

Two studies compared abbreviated MRI protocols without contrast versus full gadoxetic acid–enhanced MRI for HCC follow-up and for detection of CRLMs. In a retrospective study comparing non-contrast MRI (T1W, T2W, and DWI) versus gadoxetic acid–enhanced MRI in 483 patients with HCC undergoing follow-up post hepatectomy [19], full gadoxetic acid–enhanced MRI provided significantly superior sensitivity (99% vs. 95%; *p* = 0.025) and accuracy (99% vs. 98%; *p =* 0.021) compared with non-contrast abbreviated MRI. In this study, a low-risk patient cohort followed for ≥ 1 year without HCC recurrence, non-contrast MRI did not differ significantly in diagnostic performance to gadoxetic acid–enhanced MRI at 1–2 years (sensitivity: 89% vs. 100%, *n* = 94) and ≥ 2 years post surgery (100%, both techniques; *n* = 29), suggesting that non-contrast MRI could replace full MRI in a patient subgroup at low risk of recurrence [19]. Hwang et al retrospectively compared non-contrast MRI (T1W, T2W, and DWI), with or without contrast-enhanced computed tomography (CECT), versus gadoxetic acid–enhanced MRI in patients with colorectal cancer [20]. There were no significant differences between the techniques in sensitivity, specificity, NPV, and positive predictive value (PPV) for all lesions, lesions ≤ 1.0 cm, and lesions > 1.0 cm. The authors concluded that non-contrast abbreviated MRI could be an alternative to contrast-enhanced MRI, at least in patients with a relatively high risk of CRLMs.

In conclusion, this is a relatively nascent topic. The few publications available are retrospective and likely to over-estimate sensitivity and cost savings. Components of the abbreviated protocols also varied between these studies. During the discussion at the Liver Forum, some delegates recommended that an abbreviated gadoxetic acid–enhanced MRI protocol should include HBP plus DWI, while inclusion of T2W sequence(s) was optional.

- *Consensus statement #4*
- Abbreviated gadoxetic acid–enhanced MRI protocols can be considered a method of screening and surveillance for HCC in at-risk patients, with the caveat that there is appropriate expertise available (44/80; 55% agreement. Consensus was not reached).
- *Notes:* Insufficient evidence is available for recommending the frequency of MRI surveillance (annual vs. biannual) or the method to assess diagnostic performance (per lesion/per patient). The cost-effectiveness of this approach may be possible to establish, but is dependent on reimbursement levels and the risk for developing HCC. Standardized criteria for imaging features in an abbreviated protocol should be developed*.*
- *Consensus statement #5*
- Abbreviated gadoxetic acid–enhanced MRI can be considered a method for follow-up in patients at risk of or with known colorectal liver metastases, with the caveat that a full staging method of imaging is required at baseline (45/76; 59% agreement. Consensus was not reached).

## Evaluation of treatment response of liver metastases and clinical impact

### Response prediction and assessing response to systemic therapy

Pre-operative chemotherapy is commonly used in patients with resectable liver metastatic disease to improve survival [21, 22], although the benefit of this intervention remains equivocal [23, 24]. Radiologic response to pre-operative chemotherapy is associated with better survival post-resection [25]. Thus, accurate predictors of response at baseline imaging are desired. Furthermore, the Response Evaluation Criteria in Solid Tumors (RECIST 1.1) is recognized to have shortcomings using size measurements only to assess response [26, 27]. In consequence, novel functional and morphologic imaging parameters are being investigated.

### Prediction of therapy response in baseline MRI

Murata et al performed a retrospective study to assess whether gadoxetic acid–enhanced MRI could predict response to chemotherapy in patients with CRLMs [28]. Relative tumor enhancement compared with surrounding liver parenchyma, measured in the HBP, was higher in responders than non-responders (37.2% ± 10.9% vs. 17.9% ± 10.5%, respectively; *p* = < 0.001). Using a relative tumor enhancement cut-off value of 24.2%, the sensitivity and specificity for detection of responders were 93.3% and 72.7%, respectively. The authors hypothesized that OATP1B3 expression on tumors and gadoxetic acid uptake may be associated with chemotherapeutic response.

Another retrospective study assessed a scoring system based on three gadoxetic acid–enhanced MRI features—overall heterogeneity, tumor–liver interface, and peripheral rim enhancement—to predict response to chemotherapy in patients with CRLMs [29]. On multiple-regression analysis, residual vital tumor (the primary outcome) was statistically associated with the scoring system (*p* < 0.001), chemotherapy response group (*p* < 0.001), and apparent diffusion coefficient (ADC) (*p* < 0.021).

### Response assessment beyond RECIST

Hosseini-Nik et al performed gadoxetic acid–enhanced MRI, including DWI after chemotherapy in patients scheduled for liver resection to derive ADC, normalized relative enhancement, and relative signal intensity difference (RSID) for liver parenchyma versus metastases [30]. Patients who showed a complete response at post-surgical histopathology had significantly higher ADC (*p* = 0.03) and lower RSID (*p* = 0.008) than patients with partial response. Combination of these indices (i.e., ADC = 1.25–1.9 × 10^-3^ mm^2^/s, normalized relative enhancement = 0–35%, and RSID < 120) had 60% sensitivity and 100% specificity for detection of complete pathologic response.

In conclusion, gadoxetic acid–enhanced MRI shows promise for predicting and assessing the response to systemic chemotherapy.

### Pre-surgical or pre-interventional planning after systemic therapy

Imaging for pre-surgical/pre-interventional assessment should be highly sensitive for detecting metastatic burden [31]. Gadoxetic acid–enhanced MRI and CECT were prospectively compared for the pre-operative detection of 151 histologically confirmed CRLM in patients who underwent chemotherapy [31]. Gadoxetic acid–enhanced MRI had significantly higher sensitivity for detection of CRLM ≤ 1.0 cm (86% vs. 46%; *p* < 0.001), a lower rate of indeterminate diagnosis (7% vs. 33%; *p* < 0.001), and higher interobserver concordance in characterizing lesions ≤ 1.0 cm (72% vs. 51%; *p* = 0.041). The authors noted that the higher lesion yield of gadoxetic acid–enhanced MRI than CECT would have changed the surgical plan in 45% of patients.

“Disappearing” liver metastases (DLMs) disappear or become occult on imaging after pre-operative chemotherapy. In general, factors pre-disposing to DLMs are small baseline lesion size (< 2 cm), increased number of treatment cycles, oxaliplatin-based therapy, increased number of CRLMs (≥ 3), and synchronous CRLMs [32]. Despite a complete radiologic response, however, up to 80% DLMs on CECT show microscopic residual disease at the anatomic location and 60–74% DLMs re-appear in situ [33, 34]. Due to a higher lesion detection rate of MRI compared to CECT and a lower impairment of the sensitivity of MRI by therapy-induced changes, pre-operative contrast-enhanced MRI (and particularly gadoxetic acid–enhanced MRI) show a higher sensitivity for correct DLM assessment [32]. The outcomes of three retrospective studies reporting the role of gadoxetic acid–enhanced MRI to assess DLMs are summarized in Table 1.

**Table 1:** Studies describing gadoxetic acid–enhanced MRI to assess DLMs

Author	Study design	Outcome	Conclusion
Park et al 2017 [35]	Retrospective comparison: gadoxetic acid–enhanced MRI vs. CECT for prediction of true absence of tumor within 1 year post surgery (*n* = 87 patients, 393 CRLMs)	True absence of tumor shown at pathology in 97/393 CRLMs (25%) Positive predictive values: • Gadoxetic acid–enhanced MRI: 78% • CECT: 35% (*p* < 0.001 vs. gadoxetic acid–enhanced MRI)	Gadoxetic acid–enhanced MRI is superior to CECT for post-chemotherapy assessment of DLMs
Owen et al 2016 [36]	Retrospective follow-up: gadoxetic acid–enhanced MRI up to 1 year post surgery (*n* = 11 patients, 77 DLMs)	At surgical pathology or 1-year follow-up imaging: • 55% DLMs demonstrated viable tumor (*n* = 21) or recurrence (*n* = 21) • 39% DLMs were non-viable or without evidence of recurrence	Over half of viable DLMs were missed by gadoxetic acid–enhanced MRI in this small study
Kim et al 2017 [37]	Retrospective follow-up: gadoxetic acid–enhanced MRI and DWI at 1 and 2 years post chemotherapy (*n* = 43 patients, 168 DLMs and 48 RT-CRLMs (≤ 5 mm) colorectal liver metastases	At 1 and 2 years, respectively: • Cumulative in situ recurrence rates for DLM: 11% and 16% • Cumulative progression rates for RT-CLM: 27% and 33%	DLMs on gadoxetic acid–enhanced MRI and DWI indicated high possibility of complete remissions

*Abbreviations: CECT*, contrast-enhanced computed tomography; *CRLM*, colorectal liver metastasis; *DLM*, disappearing liver metastasis; *DWI*, diffusion-weighted imaging; *RT-CRLM*, residual tiny colorectal liver metastasis

Sinusoidal obstruction syndrome (SOS) is an adverse effect of pre-operative systemic chemotherapy in CRLM, with a reported incidence of 42–51%. SOS can lead to diffuse hepatopathy, focal hepatopathy, and focal nodular hyperplasia-like nodules [38], which is associated with higher post-operative morbidity and mortality. Gadoxetic acid–enhanced MRI typically identifies SOS as a diffuse hypointensity on HBP imaging, with a high specificity (96–100%) and good interobserver agreement [39]. This led the European Society of Gastrointestinal and Abdominal Radiology to recommend gadoxetic acid–enhanced MRI for the diagnosis of SOS in patients with chemotherapy-treated CRLM [40].

- *Consensus statement #6*
- Pre-surgical or pre-interventional planning of liver metastases after systemic therapy is best assessed with gadoxetic acid–enhanced MRI (65/77; 84% agreement).
- *Consensus statement #7*
- Gadoxetic acid–enhanced MRI (including DWI) is superior to computed tomography (CT) for the assessment of disappearing liver metastases from colorectal cancer after systemic therapy, although the disappearance of liver metastases on gadoxetic acid–enhanced MRI does not indicate a complete pathologic response (77/81; 95% agreement).

## International diagnostic HCC guidelines and LI-RADS

Imaging-based diagnostic systems for HCC have been published by numerous specialist societies [41]. Notably, all recent guidelines included gadoxetic acid–enhanced MRI in their diagnostic algorithms [6, 42–47].

There is a lack of standardization across HCC guidelines on the target populations requiring surveillance, diagnosis, staging, or monitoring; the imaging modalities and imaging criteria adopted; and treatment practices [48]. These differences are illustrated in Table 2, which compares the imaging components used for the diagnosis of HCC by five international guidelines. Essentially, there is not complete agreement in the use of any imaging components between these guidelines.

**Table 2:** HCC imaging systems: similarities and differences in guidelines. Adapted from [41, 43]

Component	American Association for the Study of Liver Diseases	Asian Pacific Association for the Study of the Liver	European Association for the Study of the Liver	Japan Society of Hepatology	Korean Liver Cancer Association-National Cancer Center
“APHE”	Non-rim APHE	APHE	Non-rim APHE	APHE	Non-targetoid APHE
“Washout” on gadoxetic acid–enhanced MRI	PVP only	PVP (and HBP)	PVP only	PVP, HBP	PVP, TP, HBP
CEUS as second modality	No	Yes (Sonazoid)	Yes	Yes (Sonazoid)	Yes
Diagnosis category	LR-1~5, LR-M	HCC	HCC	HCC	HCC, probable HCC, indeterminate
Image criteria for < 1-cm HCC	No	Yes	No	No	No
Capsule as a major criterion	Yes	No	No	No	No
“Interval growth” as a major criterion	Yes	No	No	No	No

*Abbreviations: APHE*, arterial phase hyperenhancement; *CEUS*, contrast-enhanced ultrasound; *HBP*, hepatobiliary phase; *HCC*, hepatocellular carcinoma; *PVP*, portovenous phase; *TP*, transitional phase

There is a notable divergence in treatment approaches between the North American/European guidelines and those from Asia. In North America and Europe, the diagnostic criteria are designed to achieve *high specificity* for the diagnosis of definite HCC (“the liver transplant setting”). In Asia, by contrast, diagnostic criteria favor *high sensitivity* for the detection of early-stage HCC (“the local treatment setting”) [41].

Standardization of imaging criteria and treatment practices across HCC guidelines represents a long-term objective—although the recent integration of LI-RADS into AASLD practice guidelines represents an early step to achieving this goal [5]. Standardization of terminology across HCC guidelines is a more achievable objective. This would encourage the development of registries and sharing of scientific data between centers, while in the clinical practice setting, it would reduce ambiguities or inaccurate communication [49]. LI-RADS has been developing a lexicon for definitions and reporting since 2011 and will continue to refine this in future updates [50].

- *Consensus statement #8*
- To facilitate research, enable meta-analysis, and improve patient care, international guidelines should adopt the LI-RADS terminology and recommend the use of standardized reporting for the radiologic diagnosis of HCC (60/71; 85% agreement).

*Caveat:* This consensus statement relates to LI-RADS terms and definitions, not to LI-RADS diagnostic criteria and categories*.* Thus, radiologists and other specialists caring for patients with liver disease are encouraged to use the LI-RADS terms and definitions for clinical care and publications, even if a LI-RADS algorithm is not applied.

## Gadoxetic acid–enhanced MRI for staging, treatment decision-making, and follow-up of HCC

### Treatment options

The Barcelona Clinic Liver Cancer (BCLC) staging system is a widely used and validated algorithm that selects treatments based on tumor burden, liver function, and performance status [42]. Alternative treatment algorithms have also evolved [43, 51], primarily reflecting recent changes in the treatment strategies for intermediate HCC [52]. In all algorithms, imaging criteria underpin treatment decision-making, providing information on lesion location, number, size, and stage.

### Staging

The staging performance of gadoxetic acid–enhanced dynamic MRI and CECT was retrospectively compared in 195 patients with HCC, relative to the final BCLC staging [53]. Gadoxetic acid–enhanced MRI provided significantly greater sensitivity (91% vs. 80%; *p* < 0.0001) and more accurate BCLC staging (93% vs. 81%; *p* < 0.0001) than CECT. BCLC stage was correctly changed by gadoxetic acid–enhanced MRI in 14% (27/195) of patients who showed a difference between the CECT-derived and the final BCLC stage.

### Therapy planning (resection, transplantation, local ablation)

Lee et al assessed the ability of gadoxetic acid–enhanced MRI to predict HCC recurrence in a retrospective study of 122 patients before living donor liver transplantation [54]. Independent predictors of HCC recurrence were being “beyond the Milan criteria” (HR: 3.54; *p* = 0.030) and peritumoral hypointensity on HBP imaging (HR: 18.30; *p* < 0.001). HBP MRI had a 90% accuracy to categorize the Milan criteria when compared with pathology on the explanted liver. Peritumoral hypointensity on HBP was significantly associated with worse tumor grade (*p* = 0.01) and microvascular invasion (MVI) (*p* < 0.001).

Another retrospective study assessed whether HBP imaging, in addition to dynamic imaging, improved the diagnostic performance of gadoxetic acid–enhanced liver MRI in patients with HCC who underwent transplantation [55]. HBP imaging significantly improved sensitivity for lesion detection compared with dynamic imaging, particularly for 1–2-cm HCCs (21% vs. 45%, respectively, reader 1 [R1]; 28% vs. 41%, reader 2 [R2]). The accuracy of patient allocation based on Milan criteria also improved from 89% with gadoxetic acid–enhanced dynamic images to 92% when adding HBP images.

The presence of HBP hypointense nodules without arterial phase hyperenhancement (APHE) on pre-operative gadoxetic acid–enhanced MRI was reported by Lee et al to be a significant predictor of recurrence-free survival (RFS) after hepatic resection and radiofrequency ablation (RFA) [56]. In patients with HBP hypointense nodules without APHE, 5-year RFS was 34% after hepatic resection and 28% after RFA (*p* = 0.618). In patients without HBP hypointense nodules and APHE, 5-year RFS was superior after hepatic resection compared to RFA (65% versus 51%; *p* = 0.042), due to a lower incidence of local tumor progression post resection. The absence of HBP hypointense nodules without APHE on gadoxetic acid–enhanced MR may help to select treatment, but further research on the optimal treatment of such lesions is required.

The prospective, randomized SORAMIC trial assessed the improvement in survival from selective internal radiation therapy combined with sorafenib versus sorafenib alone in patients with advanced HCC (palliative arm), as well as assessing the improvement in time to recurrence from adjuvant sorafenib after local ablation versus local ablation alone in patients with early HCC (curative arm). A SORAMIC substudy compared the accuracy of baseline gadoxetic acid–enhanced MRI, using criteria developed by Renzulli et al [57], relative to multi-slice CECT and dynamic MRI, using the European Association for the Study of the Liver (EASL) criteria, for stratifying patients to palliative or curative treatment [58]. Gadoxetic acid–enhanced MRI provided superior accuracy for treatment decision-making (83% and 81%, respectively, R1 and R2; intent-to-treat population *n* = 530; *p* < 0.001) compared with CECT (74% and 71%) and dynamic MRI (76% and 70%, respectively).

### Follow-up criteria

The RECIST criteria have a number of limitations in assessing HCC response: RECIST-assessed expected tumor shrinkage can underestimate the response to therapy; the criteria demand strict requirements for patient selection and cannot be used in routine clinical practice [59]; furthermore, the RECIST criteria were designed for cytotoxic agents. With the increasing use of biologic and locoregional therapies (LRT), assessment of tumor size has a limited role for response assessment [60]. The modified RECIST (mRECIST), EASL, and LI-RADS use contrast-enhanced images that correlate more accurately with residual disease burden and survival in patients treated with ablation, transarterial chemoembolization, and radioembolization [6, 61–65].

Gordic et al compared RECIST, mRECIST, EASL, degree of tumor necrosis on subtraction MRI, and DWI for their ability to predict complete pathologic necrosis in patients with HCC undergoing liver transplantation after LRT for bridging [66]. EASL, mRECIST, dynamic phase subtraction images, and qualitative DWI were significant predictors of complete pathologic necrosis (*p* < 0.001), while RECIST and ADC were not. Subtraction showed the strongest correlation with pathologic degree of tumor necrosis (*r =* 0.71–0.72; *p* < 0.0001) and was recommended by the authors for assessing HCC response to LRT when using MRI.

### Follow-up post-ablation and resection

In patients at risk of early HCC recurrence, accurate early diagnosis may help to select patients for salvage therapy. Rimola et al prospectively followed 34 patients with HCC who had a complete response to resection and/or ablation, in whom 53 new focal lesions (enhancing in the arterial phase without washout) were detected with extracellular contrast-enhanced MRI (EC-MRI) during follow-up [67]. The combination of HBP hypointensity on gadoxetic acid–enhanced MRI and hyperintensity on DWI had high specificity (91%) and PPV (96%), but limited sensitivity (55%), for the detection of HCC recurrence prior to confident diagnosis by histopathology or EC-MRI.

### Follow-up post-radioembolization

Radioembolization using β-emitting yttrium-90 microspheres is increasingly used to treat primary and metastatic liver cancers. For assessing tumor response to radioembolization, Joo et al suggest that imaging changes in size, enhancing tumor burden, and diffusion restriction together with serum tumor markers can be useful, particularly in combination [68]. Gadoxetic acid–enhanced MRI additionally provides functional information on hepatocyte uptake during treatment that may be useful for evaluating the extent of radiation effects on liver parenchyma [68].

Schelhorn et al compared gadobutrol (Gadovist/Gadavist^®^)-enhanced MRI against gadoxetic acid–enhanced MRI, with and without HBP imaging, for assessing response after radioembolization [69]. Patients with HCC underwent MRI on consecutive days before radioembolization and 30, 90, 180, and 270 days post-radioembolization. Tumor progression was confirmed in 14/82 study visits by CT combined with α-fetoprotein or γ-glutamyl transferase assessment. The sensitivity and specificity of gadoxetic acid–enhanced MRI with HBP imaging (0.929 and 0.971, respectively) were higher than gadoxetic acid–enhanced MRI without HBP imaging (0.786 and 0.941) or gadobutrol-enhanced MRI (0.643 and 0.956).

### Follow-up post chemoembolization

HCC is 6.5-fold more likely to recur in the first year post chemoembolization than in the second [70]. Reflecting this, 3-monthly imaging is recommended in the first year post treatment, with increased imaging intervals subsequently [6, 42]. There is no evidence to demonstrate the superiority of one technique (i.e., gadoxetic acid–enhanced MRI, EC-MRI, or CT) over another for assessment of response post-chemoembolization.

### Follow-up using radiogenomics and radiomics

The potential of radiomics-based approaches to predict response and survival in HCC is an area of active investigation [71, 72]. A preliminary study in 38 patients with HCC demonstrated a correlation between phenotypic MRI/CT imaging traits (including infiltrative pattern, mosaic appearance, presence of macrovascular invasion, size > 5 cm) and gene signatures for aggressive HCC [73].

Yang et al described the development of a nomogram incorporating clinic-radiologic risk factors and radiomics features derived from gadoxetic acid–enhanced MRI HBP images for the pre-operative prediction of individualized risk of MVI in patients with HCC [74].

- *Consensus statement #9*
- Evaluation of HCC response includes tumor size and degree of enhancement/necrosis based on dynamic MRI or CT, as well as assessment of new lesions (70/79; 89% agreement).
- *Consensus statement #10*
- Gadoxetic acid–enhanced MRI is accurate for the staging of HCC before and after treatment with locoregional/biologic therapies (63/71; 89% agreement).

## Future possible indications of gadoxetic acid–enhanced MRI

### Liver function

#### Chronic liver disease

Liver fibrosis is a key determinant in the natural history of chronic liver diseases. Estimating the degree of liver fibrosis is of clinical importance, because it influences the surveillance, treatment, and prognosis of disease. Gadoxetic acid uptake in the HBP is being investigated as a biomarker for liver function and staging of fibrosis and cirrhosis, as well as prediction of liver transplant graft survival and pre-operative risk assessment of liver failure after major resection [75]. Which MR-derived quantitative or semi-quantitative measures are most suitable for assessment is unresolved. The techniques summarized in Table 3 hold promise.

**Table 3:** Summary of studies describing gadoxetic acid–enhanced MRI in chronic liver disease

Author	Study design	Outcome	Conclusion
Feier et al 2016 [76]	Retrospective study to assess the diagnostic efficacy of multiparametric MRI in chronic liver disease (77 patients with Metavir fibrosis scores F0-F4)	Relative enhancement in HBP, liver:muscle ratio for SWI, and ADC measurements differed significantly among patients with different degrees of fibrosis (*p* < 0.004) Combining the three parameters, area under the curve was 94% for detecting ≥ F1, 95% for ≥ F2, 90% for ≥ F3, and 93% for F4	Multiparametric MRI is a non-invasive diagnostic tool for staging liver fibrosis
Bastati et al 2020 [77]	Retrospective study to assess functional liver imaging score derived from 20-min post-injection to predict outcomes in chronic liver disease (*n* = 265 patients)	FLIS derived from parenchymal contrast enhancement, biliary contrast excretion, and portal vein sign was independently predictive of liver decompensation during follow-up of cACLD: aHR 3.7 (*p =* 0.04) FLIS was an independent risk factor for transplant-free mortality in cACLD (aHR: 7.4) (*p* < 0.001) and decompensated ACLD (aHR 3.8) (*p =* 0.004)	FLIS identified patients with advanced chronic liver disease at increased risk for first hepatic decompensation and for mortality
Beer et al 2019 [78]	Retrospective study to assess the correlation of four HBP-based scores in patients with mixed chronic liver disease (*n* = 287 patients)	RLE, contrast uptake index, hepatic uptake index, and liver:spleen contrast index correlated significantly (*p* < 0.001) with albumin–bilirubin, MELD, and Child-Turcotte-Pugh scores and discriminated patients with a MELD score ≥ 15 vs. ≤ 14	All HBP-based parameters correlated with clinical and laboratory scores of hepatic dysfunction
Asenbaum et al 2017 [79]	Retrospective study to assess the impact of PH on liver MRI in chronic liver disease (*n* = 178 patients)	HVPG correlated inversely with RLE on 20-min gadoxetic acid–enhanced MRI (*r* = 0.18; *p* < 0.0001) On univariate analysis, clinically significant and severe PH (HVPG ≥ 10 and ≥ 12 mmHg, respectively) were associated with delayed biliary contrast excretion and PVHS (*p* < 0.01, all) Lower RLE and PVHS were associated with lower 3-year transplantation-free survival (HR: 0.98 and 3.99, respectively) (*p* = 0.002, all), independent of Child-Turcotte-Pugh and MELD scores	PVHS on gadoxetic acid–enhanced MRI is an independent indicator of severe PH and may enable more accurate diagnosis
Sandrasegaran et al 2018 [80]	Retrospective study to determine the value of quantitative parameters of gadoxetic acid–enhanced MRI in predicting prognosis in patients with cirrhosis (*n* = 63 patients)	Variceal bleeding, hepatic encephalopathy, or mortality occurred in 15, 31, and 27 patients, respectively, within 2 years ER at 15 min (ER15) and CES at 20 min (CES20) were the best MRI predictors for events Areas under the ROC curve for predicting variceal bleeding were 0.785 and 0.729, respectively, for ER15 and CES20, vs. 0.673 and 0.714, respectively, for Child-Turcotte-Pugh and MELD scores ER15 < 48 had 96% sensitivity and 84% specificity for predicting the onset of hepatic encephalopathy within 2 years	ER15 and CES20 were equivalent or better predictors of major morbidity and mortality in patients with cirrhosis compared to common clinical scores

*Abbreviations: ACLD*, advanced chronic liver disease; *ADC*, apparent diffusion coefficient; *aHR*, adjusted hazard ratio; *cACLD*, compensated advanced chronic liver disease; *CES*, contrast enhancement spleen index; *ER*, enhancement ratio; *FLIS*, functional liver imaging score; *HBP*, hepatobiliary phase; *HVPG*, hepatic venous pressure gradient; *MELD*, model of end-stage liver disease; *PH*, portal hypertension; *PVHS*, portal vein hyperintensity sign; *RLE*, relative liver enhancement; *ROC*, receiver operating characteristic; *SWI*, susceptibility-weighted imaging

#### Hepatectomy, liver transplantation

In a retrospective study with 62 patients, Asenbaum et al [81] looked at how well functional future liver remnant (functFLR), as calculated from the RLE on gadoxetic acid–enhanced MRI and volumetry on multidetector CT done within 10 weeks of a planned major resection, predicted post-hepatectomy liver failure (PHLF) following major liver resection compared to well-established clinical tests. In a multivariate analysis, the authors found that a decreased functFLR was independently associated with the probability of PHLF (0.561; *p* = 0.002). Comparing receiver operating characteristic curves, functFLR showed a significantly higher area under the curve (0.904; *p* < 0.001) than established variables. The authors concluded that functFLR seems to be superior to established variables in the prediction of PHLF after major liver resection.

Wibmer et al evaluated gadoxetic acid–enhanced MRI in liver transplant recipients with regard to graft function and mortality at 1 year from imaging [82]. Impaired HBP excretion, defined as absence of gadoxetic acid visualization in the common bile duct 20 min after injection, was identified in 20/51 patients after transplantation. The impaired excretion group had significantly higher serum bilirubin (*p* < 0.001), aspartate aminotransferase (*p* = 0.003), alkaline phosphatase (*p* = 0.007), and higher median MELD score (*p* < 0.001). Within 1 year of MRI, 55% of these 20 patients had died (*n* = 7) or underwent retransplantation (*n* = 4), while all patients with normal HBP excretion survived without retransplantation (*p* < 0.001). RLE 20 min after gadoxetic acid injection was directly related to serum cholinesterase (*p* < 0.001) and inversely related to serum bilirubin (*p* = 0.0098), aspartate aminotransferase (*p* = 0.007), and MELD score (*p* < 0.001). RLE was also directly related to the probability of 1-year retransplantation-free survival (*p* = 0.005).

### Biliary system assessment

Bile leakage is a common complication of abdominal surgical procedures and its precise localization is important for selecting optimal management [83]. A retrospective analysis in 34 patients with suspected bile leak showed that gadoxetic acid–enhanced MRI had an overall 96% sensitivity and 97% accuracy for diagnosis and location of an active bile leak [84]. Sensitivity increased in delayed HBP: from 43% for 20–25-min HBP to 93% for combined 20–25 and 60–90-min HBP, and 96% for combined 20–25, 60–90, and 150–180-min HBP.

Expanding bile leaks after blunt liver trauma require more aggressive treatment than contained leaks. The presence of expanding bile leaks was assessed by T1W MR cholangiopancreatography (MRCP) gadoxetic acid–enhanced MRC in 22 patients with recent major blunt trauma [85]. T1W MRC 30 and 90 min after gadoxetic acid administration had higher scores for biliary tree visualization and leak detection compared with 10- and 20-min acquisitions and showed an excellent interrater reliability.

T1W MRC can be useful for visualization of non-dilated duct. Biliary visualization was assessed in 29 right-liver donors using four techniques: 3D T2W MRCP, 2D T2W MRCP, breath-hold T1W hepatobiliary MRC (BH T1W MRC), and high-resolution 3D T1W hepatobiliary MR (Nav T1 MRC) using gadoxetic acid [86]. Both BH T1 MRC and Nav T1 MRC improved the accuracy of visualization and specificity of biliary diagnosis when added to 3D/2D T2W MRCP in 29 living liver donors. The Nav T1 MRC set using gadoxetic acid showed the highest diagnostic confidence and visualization scores for branching and overall ducts. In another study on living liver donors, gadoxetic acid–enhanced isotropic high-resolution (IHR) 3D T1W MRC was compared to 3D multi-slice T2W MRCP for evaluation of biliary anatomy [87]. IHR-T1W-MRC provided significantly improved visibility and sharpness of all evaluated intrahepatic bile ducts compared with 3D T2W MRCP (all *p* < 0.05), as well as higher overall image quality (*p* < 0.01). IHR-T1W-MRC also demonstrated significantly higher agreement with the reference standard than 3D T2W MRCP in bile duct variation (88% vs. 81%; *p =* 0.03) and expected bile duct openings (77% vs. 70%; *p =* 0.006).

Gadoxetic acid–enhanced MRI features were investigated for their ability to diagnose cystic fibrosis (CF)–associated liver disease (CFLD) in 50 CF patients and 40 controls [88]. Three imaging descriptors distinguished CFLD from controls: altered gallbladder morphology, periportal tracking, and periportal fat deposition. Prospective validation of this classification algorithm showed 94% sensitivity and 85% specificity for discriminating CFLD from controls. Disease severity correlated well with the imaging features.

- *Consensus statement #11*
- Gadoxetic acid uptake in the HBP may serve as a biomarker for liver function globally and segmentally, as well as assessment of liver fibrosis (64/67; 96% agreement).
- *Consensus statement #12*
- Gadoxetic acid T1W MRC can provide a functional and structural assessment of the biliary system (68/73; 93% agreement).

## Summary

Delegates at the 9^th^ International Forum debated the benefits from multidisciplinary treatment and approaches to standardizing the terminology in liver MRI, the important roles of gadoxetic acid–enhanced MRI in evaluating treatment response of liver metastases and in treatment decision-making for HCC, and the potential new indications for this imaging technique in quantifying liver and biliary system function.

## Supplementary Information

ESM 1 (DOCX 186 kb)

## Abbreviations

AASLD: American Association for the Study of Liver Diseases
ADC: Apparent diffusion coefficient
aHR: Adjusted hazard ratio
APHE: Arterial phase hyperenhancement
BCLC: Barcelona Clinic Liver Cancer
BH: Breath-hold
cACLD: Compensated advanced chronic liver disease
CECT: Contrast-enhanced computed tomography
CES: Contrast enhancement spleen index
CES20: Contrast enhancement spleen index at 20 min
CEUS: Contrast-enhanced ultrasound
CF: Cystic fibrosis
CFLD: Cystic fibrosis-associated liver disease
CRLM: Colorectal liver metastasis
CT: Computed tomography
DLM: “Disappearing” liver metastasis
DWI: Diffusion-weighted imaging
EASL: European Association for the Study of the Liver
EC-MRI: Extracellular contrast-enhanced magnetic resonance imaging
ER: Enhancement ratio
ER15: Enhancement ratio at 15 min
FLIS: Functional liver imaging score
FLR: Future liver remnant
functFLR: Functional future liver remnant
HBP: Hepatobiliary phase
HCC: Hepatocellular carcinoma
HR: Hazard ratio
HVPG: Hepatic venous pressure gradient
IHR: Isotropic high-resolution
LI-RADS: Liver Imaging Reporting and Data System
LRT: Locoregional interventional therapies
MELD: Model of end-stage liver disease
MRCP: Magnetic resonance cholangiopancreatography
mRECIST: Modified Response Evaluation Criteria in Solid Tumors
MRI: Magnetic resonance imaging
MVI: Microvascular invasion
Nav T1 MRC: High-resolution 3D T1W hepatobiliary MR
NPV: Negative predictive value
PHLF: Post-hepatectomy liver failure
PPV: Positive predictive value
PVHS: Portal vein hyperintensity sign
PVP: Portovenous phase
R1: Reader 1
R2: Reader 2
RECIST: Response Evaluation Criteria in Solid Tumors
RFA: Radiofrequency ablation
RFS: Recurrence-free survival
RLE: Relative liver enhancement
ROC: Receiver operating characteristic
RSID: Relative signal intensity difference
RT-CRLM: Residual tiny colorectal liver metastases
SOS: Sinusoidal obstruction syndrome
SSFSE: Single-shot, fast spin-echo
SWI: Susceptibility-weighted imaging
T1W: T1-weighted
T2W: T2-weighted
TP: Transitional phase
