# Supplementary material for: Consensus report from the 9th International Forum for Liver Magnetic Resonance Imaging: applications of gadoxetic acid-enhanced imaging
Source: Eur Radiol. 2021 Feb 1;31(8):5615–28. doi: 10.1007/s00330-020-07637-4 (PMC8270799; doi:10.1007/s00330-020-07637-4)
Supplement: Supplementary file 1 — (DOCX 186 kb) [file 330_2020_7637_MOESM1_ESM.docx]

**Consensus report from the 9^th^ International Forum for Liver Magnetic Resonance Imaging: applications of gadoxetic acid-enhanced imaging**

# Supplement 1: Delegate responses to the pre-meeting questionnaire

**Questions About Your Liver Imaging Practice**

1. How would you describe your practice? (%)

2. How many years' experience do you have in liver imaging?

3. How many years of experience do you have with gadoxetic acid?

**Which of the following technical advances have you implemented in your practice for gadoxetic acid-enhanced MRI? (multiple options possible)**

4. Multiple arterial phase acquisition (%)

5. Volume Interpolated GRE with high acceleration factors (e.g., CAIPIRINHA VIBE, compressed sense THRIVE or similar) (%)

6. Time-resolved sequences (e.g., TWIST / DISCO / 4D THRIVE or similar) (%)

7. Radial VIBE or similar (%)

8. Free-breathing volume interpolated GRE (e.g., GRASP VIBE or similar) (%)

9. How do you time your arterial phase? (%)

10. The vendor-recommended dose for gadoxetic acid enhanced MR imaging of the liver is 0.1 mL per kg body weight (0.025 mmol/kg). What dose of gadoxetic acid do you use in clinical practice? (%)

11. Gadoxetic acid should be administered undiluted as an intravenous bolus injection. Do you dilute gadoxetic acid in clinical practice? (%)

12. Depending on the liver function, the hepatobiliary phase of gadoxetic acid can be acquired starting at 10–20-min post injection. What delay do you use for the acquisition of the hepatobiliary phase? a) Cirrhotic (%)

b) Non-cirrhotic (%)

13. Do you avoid giving gadoxetic acid based on laboratory markers (e.g., elevated bilirubin or prothrombin levels or liver function test results)? (%)

14. The vendor-recommended injection rate for gadoxetic acid is 1–2 mL/sec. What is your usual injection rate of gadoxetic acid? (%)

**Questions Requesting your Clinical Perspective**

15. In your medical center, are all new diagnoses of HCC discussed at a tumor board before biopsy or treatment is considered? (%)

16. If yes, who participates in the tumor board? (%)

17. What scoring system do you use for diagnosis of HCC in daily (%)

18. Should non-hypervascular and hepatobiliary phase-hypointense lesions (i.e., early HCC / HGDN) be incorporated in treatment decision-making in HCC patients, in particular in those HCC patients scheduled for curative intended treatments? (%)

**How much do you agree to the following statements?**

19. When assessing treatment response of HCC, the mRECIST should be used whenever possible (%)

20. I am confident about using diffusion imaging and the Apparent Diffusion Coefficient (ADC) to assess treatment response in a) HCC (%)

b) Metastatic liver disease (%)

21. I would like to use gadoxetic acid MRI to assess a) Hepatic function (%)

b) For staging of hepatic fibrosis (%)

22. Using gadoxetic acid MRI in my clinical practice has positively changed our management of patients with liver metastases (%)

23. I would consider using an abbreviated liver imaging protocol in specific contexts for the diagnosis and/or follow-up of patients with a) HCC (%)

b) Liver metastases (%)
